# Nutrient-Sensing Ghrelin Receptor in Macrophages Modulates Bisphenol A-Induced Intestinal Inflammation in Mice

**DOI:** 10.3390/genes14071455

**Published:** 2023-07-16

**Authors:** Xiangcang Ye, Zeyu Liu, Hye Won Han, Ji Yeon Noh, Zheng Shen, Da Mi Kim, Hongying Wang, Huiping Guo, Johnathan Ballard, Andrei Golovko, Benjamin Morpurgo, Yuxiang Sun

**Affiliations:** 1 Department of Nutrition, Texas A&M University, College Station, TX 77843, USA; 2 Texas Institute for Genomic Medicine, College Station, TX 77843, USA; 3 USDA/ARS Children’s Nutrition Research Center, Department of Pediatrics, Baylor College of Medicine, Houston, TX 77030, USA

**Keywords:** ghrelin, GHSR, bisphenol A, macrophage, inflammation

## Abstract

Bisphenols are environmental toxins with endocrine disruptor activity, yet bisphenol A (BPA) and its analogs are still widely used in manufacturing plastic products. There is evidence showing that BPA elicits inflammation in humans and animals, but the target cell types of BPA are not well understood. In this study, we sought to determine BPA’s direct effect on macrophages and BPA immunotoxicity in mouse intestine. Ghrelin is an important nutrient-sensing hormone, acting through its receptor growth hormone secretagogue receptor (GHSR) to regulate metabolism and inflammation. We found that BPA promotes intestinal inflammation, showing increased infiltrating immune cells in colons and enhanced expression of *Ghsr* and pro-inflammatory cytokines and chemokines, such as *Il6* and *Ccl2*, in colonic mucosa. Moreover, we found that both long- and short-term BPA exposure elevated pro-inflammatory monocytes and macrophages in mouse peripheral blood mononuclear cells (PBMC) and peritoneal macrophages (PM), respectively. To determine the role of GHSR in BPA-mediated inflammation, we generated *Ghsr* deletion mutation in murine macrophage RAW264.7 using CRISPR gene editing. In wild-type RAW264.7 cells, the BPA exposure promotes macrophage pro-inflammatory polarization and increases *Ghsr* and cytokine/chemokine *Il6* and *Ccl2* expression. Interestingly, *Ghsr* deletion mutants showed a marked reduction in pro-inflammatory cytokine/chemokine expression in response to BPA, suggesting that GHSR is required for the BPA-induced pro-inflammatory response. Further understanding how nutrient-sensing GHSR signaling regulates BPA intestinal immunotoxicity will help design new strategies to mitigate BPA immunotoxicity and provide policy guidance for BPA biosafety.

## 1. Introduction

Inflammatory bowel disease (IBD) is a debilitating disease that severely decreases the quality of life. Epidemiological studies show that IBD continues to grow globally [1,2]. IBD is a chronic inflammatory condition with recurrent episodes of diarrhea, bloody stool and weight loss. Two major types of IBD are Crohn’s disease and ulcerative colitis. Current treatments for IBD are primarily for intestinal inflammation control and symptom management. The cause of IBD includes chronic exposure to environmental chemical pollutants, which have been linked to IBD pathogenesis because of their detrimental effects on genetic/epigenetic factors, microbiota, and immune functions [3,4,5].

Bisphenol A (BPA) is one of the most significant environmental pollutants in modern times. It is detectable in over 90% of human urine samples [6,7,8]. BPA is a monomeric precursor of polycarbonate plastics and epoxy resins, which are widely used in manufacturing plastic goods such as water bottles, food containers, toys, and medical devices. Particularly, high temperature accelerates BPA leaching from plasticware into water and food that eventually enters the human body. Studies showed that BPA is an endocrine-disrupting chemical, primarily targeting estrogen receptors [9], which not only has an impact on the reproductive system but also is linked to inflammatory disorders, such as IBD [10,11], obesity [12,13], and diabetes [14,15]. Recent evidence showed that BPA and its analogs disturb intestinal homeostasis in mice [16,17,18,19].

Macrophages are the gatekeepers of intestinal immunity. Specifically, the macrophages in lamina propria play a crucial role in intestinal homeostasis and inflammation [20,21]. In mice with colitis, pro-inflammatory Ly6C^high^ monocytes massively invade the colon mucosa and differentiate into inflammatory macrophages [22]. Similarly, human CD14^+^ inflammatory macrophages are prevalent in the colon mucosa of IBD patients [20,23]. Emerging evidence shows that macrophages have critical roles in intestinal inflammation and resolution. The dysfunction of macrophages is linked to persistent inflammation in the intestinal mucosa [21]. Although there were early studies on myeloid cell activity and the relationship with BPA exposure [24,25,26], it is not still clear whether their interactions contribute to intestinal inflammation.

Orexigenic and anorexigenic peptide hormones such as ghrelin and leptin regulate the human body’s energy homeostasis and cellular metabolism. The growth hormone secretagogue receptor (GHSR) is a functional receptor of ghrelin, but GHSR, as a G protein-coupled receptor, has a high level of constitutive activity that is independent of ghrelin binding [27,28]. The highest expression of the *Ghsr* gene is detected in certain neurons of the hypothalamus; it is also readily detectable in mature macrophages [29,30] and other immune cells [31,32,33]. We and others have shown that GHSR and ghrelin have a functional role in modulating macrophage activation in adipose tissue inflammation and regulating pro-inflammatory cytokine release in response to the lipopolysaccharide challenge [29,30]. However, it is unknown whether the macrophage GHSR signaling is involved in the modulation of BPA immunotoxicity.

In this study, we examined the BPA effect on mouse intestinal inflammation, myeloid cell activation, and gene expression in colon mucosa. We also analyzed the BPA effects on macrophage and monocyte polarization in peripheral blood and the peritoneal cavity. Based on our observations of increased *Ghsr* expression and pro-inflammatory cytokine and chemokine gene expression in response to BPA exposure, we further assessed the effect of *Ghsr* in BPA-mediated response in macrophages. We generated *Ghsr* deletion mutants from RAW264.7 cells by CRISPR gene editing and confirmed that the GHSR function plays an important role in the modulation of BPA-induced pro-inflammatory polarization in macrophages.

## 2. Materials and Methods

### 2.1. Animals

Mice used in this study were 3–4-month-old females from C57BL/6J backgrounds. The mice were housed in a pathogen-free Laboratory Animal Resources and Research facility at Texas A&M University and were maintained under the controlled temperature and lighting cycle (23 ± 1 °C; 12 h-to-12 h light-dark cycle), with free access to purified drinking water in glass bottles and a purified rodent diet (2020X Teklad, Envigo, Indianapolis, IN, USA) that minimizes phytoestrogen by excluding the soy component. All experiments were performed under an animal use protocol approved by the Institutional Animal Care and Use Committee at Texas A&M University.

**Figure 1:**
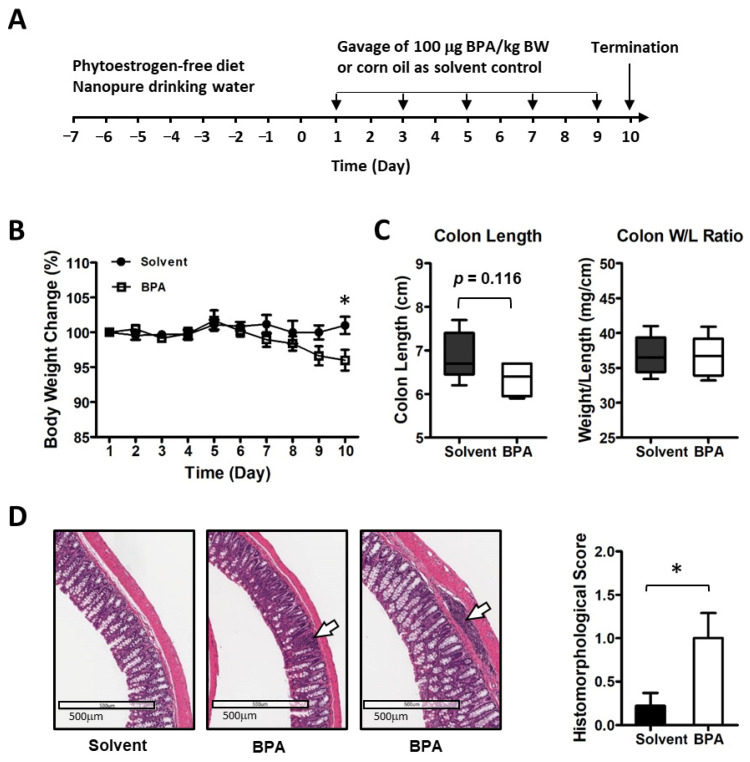
BPA elicits the inflammatory response in mouse colon mucosa. (**A**) Mice were exposed to 100 μg BPA/kg body weight by gavage on alternate days for 10 days. (**B**) BPA exposure led to a reduction in body weight. (**C**) BPA seemed to have a certain effect on colon structure. (**D**) Histo-morphological analysis of mouse colon mucosa showed increased focal inflammation (images shown at middle and right; control at left). The arrows indicate inflammatory cell infiltration in mucosal and submucosal. Mouse group *n* = 5–9. Data are reported as mean ± SEM. *: *p* < 0.05.

Mice with similar body weights and compositions were randomized and divided into experimental and control groups. BPA (Sigma-Aldrich, St. Louis, MO, USA) was first dissolved in ethanol and then diluted (1:10,000) with corn oil (Sigma-Aldrich, St. Louis, MO, USA) to form a 10 μg/mL solution, which allows the gavage volume to be adjusted proportionally to body weight (BW), e.g., administrating 250 μL BPA solution to a 25 g mouse gives a dose of 100 μg BPA/kg BW. In the study, mice were gavaged with 100 μg BPA/kg BW on alternative days (Figure 1A). Corn oil of solvent control was administrated with the same volume/BW ratio to control mice. To evaluate the disease activity index (DAI) of intestinal inflammation and colitis development, the mouse body weight, fecal consistency, and rectal bleeding were monitored and scored according to the criteria described previously [16]. Briefly, body weight change was scored as: 0: body weight gain or 0–1% loss, 1: 1–5% loss, 2: 5–10% loss, 3: 10–15% loss, 4: >15% loss compared to day 0 of the study. Fecal consistency was scored as: 0: normal stool, 1: soft but formed pellet, 2: very soft pellet, 3: diarrhea (no pellet), or 4: dysenteric diarrhea (blood in diarrhea). Rectal bleeding was scored as: 0: no bleeding, 2: the presence of visible blood in the stool (red/dark pellet), and 4: gross macroscopic bleeding (blood around the anus). To assess the acute response from BPA exposure, the experimental mice in a separate study were treated with 100 μg BPA/kg BW for 12 h. The mouse blood, peritoneal fluid, and intestine tissues were collected for analysis.

### 2.2. Flow Cytometry Analysis

Mouse peritoneal macrophages (PM) and peripheral blood mononuclear cells (PBMC) were harvested from the experimental mice according to their respective protocols [34,35]. The isolated PM and PBMC were processed for cell staining on ice and room temperature, respectively. Briefly, for PM staining, the cells were stained with a fixable LIVE/DEAD Aqua dye (Thermo Fisher, Waltham, MA, USA) and Fcγ II/III was blocked by a rat anti-mouse CD16/CD32 antibody (BD Bioscience, San Jose, CA, USA). After washing, the PM cell suspension was incubated for 30 min in the dark with an antibody cocktail, including eFluor450 anti-CD45, PE-Cy7 anti-F4/80, APC-Cy7 anti-CD11b, BV750 anti-CD38, PerCP anti-Ly6G and BV650 anti-CD206 (Table S1). After washing, the cells were fixed with 4% paraformaldehyde and permeabilized with Perm/Wash buffer (BD Bioscience, San Jose, CA, USA). Intracellular staining was conducted using antibodies including AF488 anti-iNOS, PE anti-IL1β, and PE/Dazzle 594 anti-TNFα. For PBMC staining, the antibody cocktail included BV510 anti-CD45, PE anti CD11b, eFluor-450 anti Ly6C, BV785 anti-Ly6G, APC anti-CD115, BV650 anti-CX3CR1, and FITC anti-CCR2 (Table S1). Antibody staining was followed by red blood cell lysis using BD FACS^TM^ Lysing solution (BD Bioscience, San Jose, CA, USA). Flow cytometry data were collected on Aurora full spectrum cytometry (Cytek, Tulsa, OK, USA) with a four-laser configuration. The data were further analyzed using FlowJo software (FlowJo, Ashland, OR, USA).

### 2.3. Histology

Mouse colon tissue “Swiss-rolls” were fixed in 4% paraformaldehyde for 4 h on ice, then processed with stepwise washings to the final step with 70% ethanol. The fixed tissue samples were dehydrated and embedded in paraffin according to standard histological procedures [36]. The tissue sections were then mounted on glass slides. Subsequently, after dewaxing and rehydration, the slides were stained with hematoxylin and eosin. The histological images were obtained using a Leica Aperio CS2 scanner with the software eSlideManager (Leica Biosystems, Vista, CA, USA). Histomorphological scoring of intestinal inflammation and the lesion was performed according to guidelines described by Erben et al. [37].

### 2.4. Generation of Ghsr Deletion Mutant in RAW264.7 Cells

A RAW264.7 cell line was obtained from ATCC (Manassas, VA, USA) and grown in RPMI 1640 medium supplemented with 10% fetal bovine serum (Sigma, St. Louis, MO, USA) and 10 mL/L of penicillin-streptomycin solution (Sigma, St. Louis, MO, USA). The cells were maintained at 37 °C in a humidified cell culture incubator with 5% CO_2_. To generate a *Ghsr* deletion mutant, an electroporation-based CRISPR/Cas9 gene-editing method was used [38]. The guide RNAs for conducting *Ghsr* deletion on the mouse genome were designed using an algorithm from Synthego (Redwood City, CA, USA) and synthesized as modified sgRNA (Table S2). Cas9 protein was obtained from Synthego. Electroporation of the RAW264.7 cell line with ribonucleoproteins was performed on a Gene Pulser Xcell (BioRad, Hercules, CA, USA) with BTX buffer (BTX cat# 45-0802) and exponential conditions. Concentrations were as follows: 4 μM Cas9, 4 μM sgRNA. Square-wave conditions were also used for the electroporation of the linearized plasmid. The isolation of single cells from a knockout cell pool was accomplished through limiting dilution and clonal expansion according to Synthego’s protocols, in which the cells from each edited population were diluted to 0.5–1 cells per 100 μL and plated on at least two 96-well plates. For genotyping, primers were designed to amplify the regions encompassing the edited region. The expected band sizes were 740 bp (Wt) or 150–700 bp (CRISPR del) (Table S2; Figure S1). PCR was performed using LongAmp™ Taq Master Mix (New England Biolabs, Ipswich, MA, USA) under a standard PCR condition. The PCR products were purified with a PCR Clean-Up System kit according to the manufacturer’s instructions. Sanger sequencing was performed in a Texas A&M Sequencing Core facility. The entire regions encompassing the guide cleavage sites were amplified to assess for genetic modifications.

### 2.5. Macrophage Culture and Treatment

For gene expression assay, RAW264.7 parental cells and *Ghsr* mutants were seeded with a density of 1 × 10^4^ cells/cm^2^ in six-well culture dishes. After 24 h of culturing and replenishing the RPMI medium, the cells were treated with 1 μg/mL lipopolysaccharide (LPS, Sigma, St. Louis, MO, USA) for 4 h for macrophage activation. In separate experiments, the RAW264.7 parental cells and *Ghsr* mutants were treated with 100 nM BPA (Sigma, St. Louis, MO, USA) for 4 h. The macrophages were harvested for RNA isolation or preserved in TriZol reagent (Sigma, St. Louis, MO, USA) in a −80 °C freezer.

Mouse PM were isolated from mice according to standard protocols [34]. The PM primary cells were grown in the RPMI medium containing 10 ng/mL recombinant murine macrophage colony stimulating factor (M-CSF) (PeproTech, Cranbury, NJ, USA). The BPA treatment scheme was the same as for RAW264.7 cells.

### 2.6. Real-Time Quantitative PCR

Total RNA was extracted from tissues or macrophages using Aurum™ Total RNA Mini Kit (BioRad, Hercules, CA, USA). First-strand complementary DNA (cDNA) was synthesized with iScript™ Reverse Transcription Supermix (BioRad, Hercules, CA, USA). Quantitative PCR reactions were set up using SsoAdvanced Universal SYBR^®^Green Supermix (BioRad, Hercules, CA, USA) and performed on a CFX T100 Thermocycler (BioRad, Hercules, CA, USA). Alternatively, the reactions were set up with PowerUp™ SYBR™ Green Master Mix (Applied Biosystems, Foster City, CA, USA) and performed on a LightCycler 480 II (Roche, Indianapolis, IN, USA). The reactions were run with a thermo-cycling program: UDG activation for 120 s at 50 °C (for PowerUp Mix only), activation for 120 s at 95 °C, followed by 40 cycles of denaturing 15 s at 95 °C and annealing/extension for 30 s at 60 °C. The qPCR primers sequences are listed in Supplementary Table S2.

### 2.7. Statistical Analysis

Statistical analyses were performed using ANOVA (GraphPad Software, San Diego, CA, USA) for determining the quantitative variables between the means of multiple groups and using the Student’s *t*-test for determining the two-tailed distribution and variation. Data are presented as the mean ± standard error of the mean. A *p*-value of <0.05 was considered statistically significant.

## 3. Results

### 3.1. BPA Induces Inflammatory Responses in Colonic Mucosa of Mice

In this study, we examined the BPA effect on intestinal inflammation in a 10-day exposure schedule (Figure 1A). To minimize the influence from diet phytoestrogen and the background BPA contamination, the mice were pre-conditioned with a soy-free diet and nanopure drinking water. BPA was provided in a corn oil formulation for gavage, dosed at 100 μg/kg BW at alternative days, which is higher than the Environmental Protection Agency (EPA) established guideline for human safety reference dose (50 μg/kg BW/day) but lower than the EPA’s no observed adverse effect level (NOAEL, 5 mg/kg BW/day) and the lowest observed adverse effect level (LOAEL, 50 mg/kg BW/day) [39]. We found that consecutive exposure with 100 μg BPA/kg BW in mice for 10 days resulted in an average 4.9% decrease in body weight (Figure 1B). Colon length showed a trend of decrease but colon weight to length ratio did not change (Figure 1C). A histological analysis of mouse intestine tissues showed focal inflammation with increased immune cell infiltration in colon mucosa in the BPA-treated group (Figure 1D).

**Figure 2:**
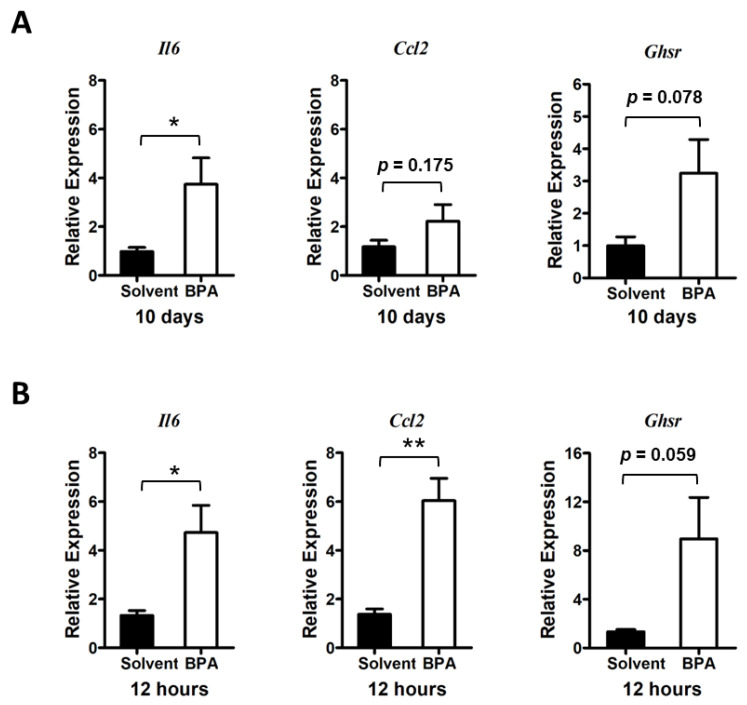
BPA exposure stimulates pro-inflammatory cytokine gene expression and GHSR expression in mouse colon mucosa. (**A**) Long treatment: Mice were exposed to BPA for 10 days by gavage of 100 μg BPA/kg BW on alternative days. Gene expression in the mouse colon mucosa was analyzed by qPCR assays, which showed an increased expression of *Il6* and *Ccl2* as well as increased expression of *Ghsr*. (**B**) Short treatment: Analysis of colon mucosal gene expression from mice were given 100 μg BPA/kg body weight for one time and sacrificed 12 h later showed an increased expression of *Il6*, *Ccl2*, and *Ghsr*. Mouse group *n* = 4–5. Data are reported as mean ± SEM. *: *p* < 0.05; **: *p* < 0.01.

Using qPCR analysis, we found that 10 days of consecutive exposure to BPA stimulated the expression of pro-inflammatory cytokine and chemokine genes, such as interleukin 6 (*Il6*) and C-C motif chemokine ligand 2 (*Ccl2*), in mouse colon mucosa (Figure 2A). As a matter of the fact, we found a more pronounced elevation of *Il6* and *Ccl2* gene expression in the colon mucosa 12 h after BPA treatment, suggesting that the pro-inflammatory effect of BPA was rapid and potentially sustainable when chronically exposed to BPA.

To assess whether GHSR signaling plays a role in regulation of the BPA-induced inflammatory response, we analyzed the *Ghsr* expression in the colon mucosa. Results showed a trend of *Ghsr* expression elevation in colon mucosa in both chronic and acute BPA exposure (Figure 2A,B), which was concurrent with increased pro-inflammatory cytokine and chemokine gene expression in the mouse colon mucosa.

### 3.2. BPA Exposure Induces Systemic Activation of Monocytes and Macrophages

**Figure 3:**
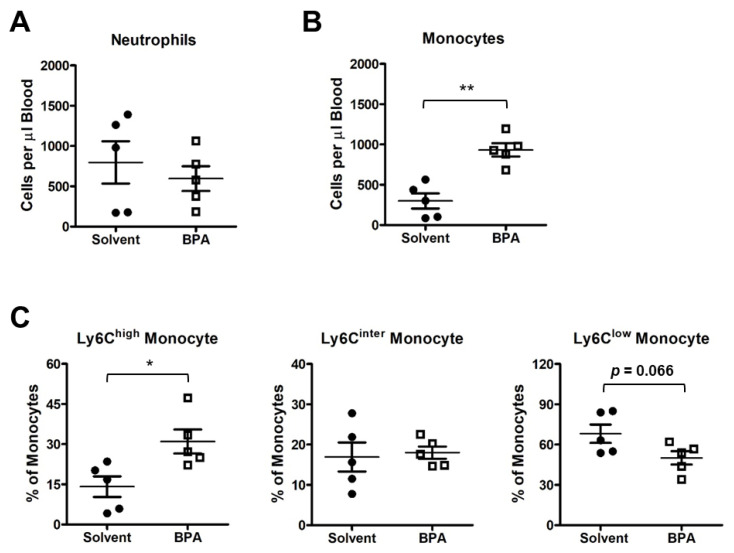
BPA exposure activates mouse peripheral blood mononuclear cells. PBMC samples were isolated from the mice exposed to one time 100 μg BPA/kg body weight, and BPMC was collected 12 h after BPA retreatment. (**A**) Flow cytometry analysis showed that the BPA exposure did not affect neutrophil abundance in blood circulation. (**B**) BPA elevated the total count of monocytes. (**C**) Ly6C^high^ pro-inflammatory monocytes were increased in response to BPA treatment, while Ly6C^inter^ patrolling monocytes and Ly6C^low^ tissue repair competent monocytes were not changed significantly. Mouse group *n* = 5. Data are reported as mean ± SEM. *: *p* < 0.05; **: *p* < 0.01.

Monocytes and macrophages are among the first responders in the body to defend against invasive microbial and environmental insults. To determine how the monocytes/macrophages responded to BPA, we isolated the leukocytes from the BPA-exposed mouse blood and analyzed the peripheral blood mononuclear cells (PBMC) by flow cytometry. We found that the monocytes were increased in the blood samples of the BPA-treated mice, while neutrophils remained unchanged (Figure 3A,B). Importantly, the pro-inflammatory monocytes with cell surface marker Ly6C^high^ were significantly increased in the PBMC population, while the Ly6C^inter^ monocytes and the patrolling anti-inflammatory Ly6C^low^ monocytes were unchanged or showed a decreasing trend under BPA treatment (Figure 3C).

**Figure 4:**
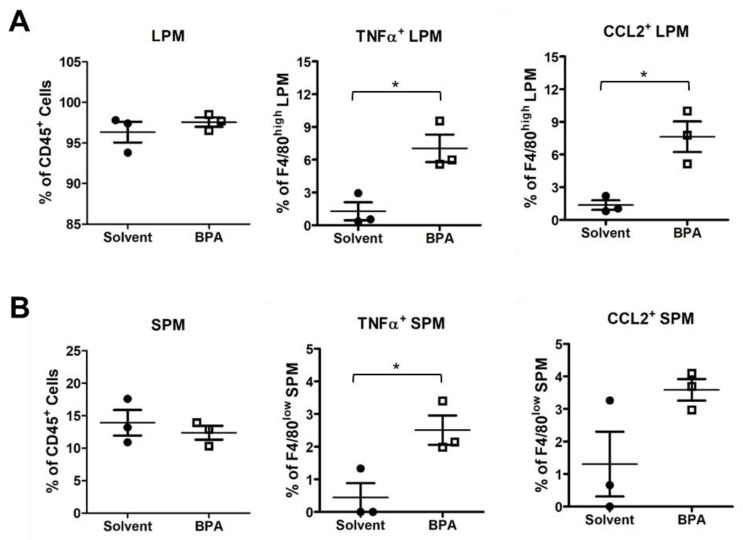
BPA enhanced pro-inflammatory marker expression in mouse peritoneal macrophages (PM). PM samples were isolated from the mice 12 h after gavages of 100 μg BPA/kg BW and analyzed by flow cytometry. (**A**) The BPA exposure did not affect total large PM (LPM) but increased the relative abundance of pro-inflammatory TNFα^+^ and CCL2^+^ LPM subpopulations. (**B**) Similarly, BPA increased TNFα^+^ and CCL2^+^ in small PM (SPM). Mouse group *n* = 3. Data are reported as mean ± SEM. *: *p* < 0.05.

Moreover, we examined the BPA effect on peritoneal macrophages (PM) by flow cytometry. PM can be separated into large PM (LPM) and small PM (SPM), according to cell surface markers F4/80 and major histocompatibility complex (MHC). LPM and SPM are derived, respectively, from embryonic precursors and adult bone marrow-derived monocytes with functions of patrolling and defense against pathogens in the peritoneal cavity [40]. In our study, BPA did not change the relative abundance of LPM nor SPM. Instead, the BPA exposure increased pro-inflammatory TNFα^+^ and CCL2^+^ subpopulations in both LPM and SPM (Figure 4A,B). These results indicate that BPA has a systemic effect on myeloid cells to induce the functional polarization of monocytes and macrophages.

**Figure 5:**
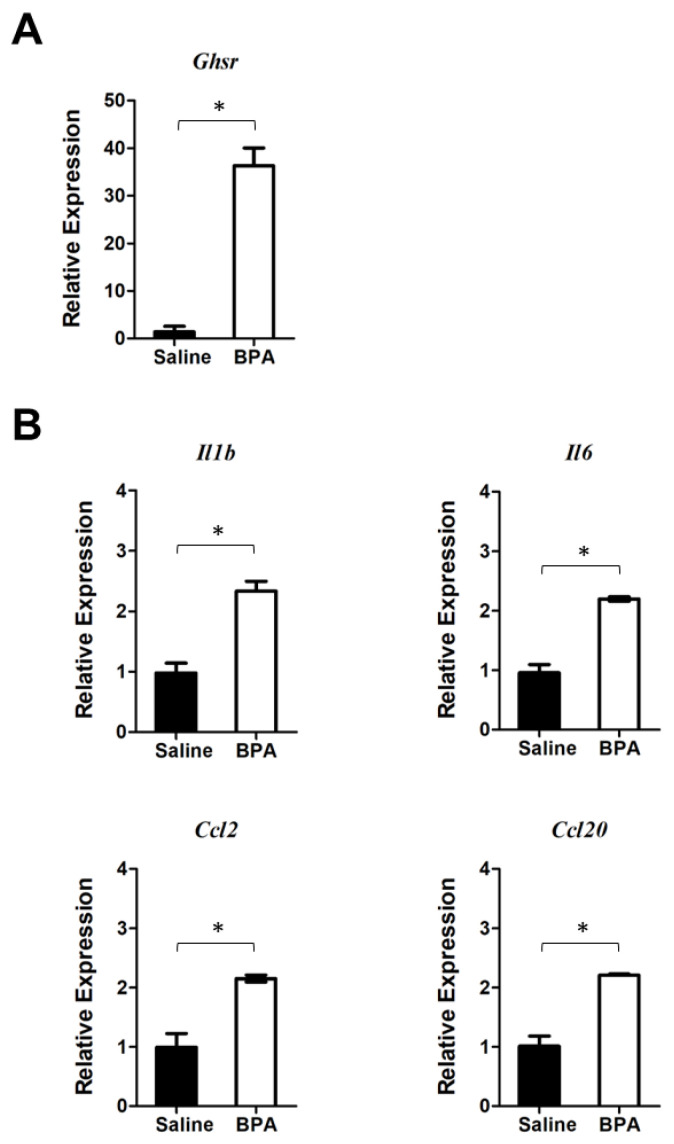
BPA stimulates *Ghsr* and pro-inflammatory cytokine and chemokine gene expression in peritoneal macrophages (PM). PM samples were harvested from the mice without pre-exposure to BPA. The PM cells were cultured overnight and then treated with 100 nM BPA for 4 h. (**A**) qPCR analysis showed that BPA induced *Ghsr* expression in PM. (**B**) BPA also elevates the expression of pro-inflammatory cytokine and chemokine genes, including *Il1b*, *Il6*, *Ccl2*, and *Ccl20* in PM. Data are reported as mean ± SEM. *: *p* < 0.05.

We further defined the direct effect of BPA by treating PM with BPA ex vivo and analyzed the gene expression of PM by qPCR analysis. Results showed that BPA markedly induced *Ghsr* expression (Figure 5A). At the same time, BPA stimulated the expression of pro-inflammatory cytokines *Il1b* and *Il6* as well as chemokines *Ccl2* and *Ccl20* in PM (Figure 5B). These results were similar to what we observed in mouse colon mucosa gene expression analysis (Figure 2), with both *Ghsr* expression and pro-inflammatory cytokine gene expression being up-regulated by BPA, suggesting that GHSR may be directly involved in the BPA-induced pro-inflammatory response in macrophages.

### 3.3. Ghsr Is Required for Pro-Inflammatory Activation of Macrophages

**Figure 6:**
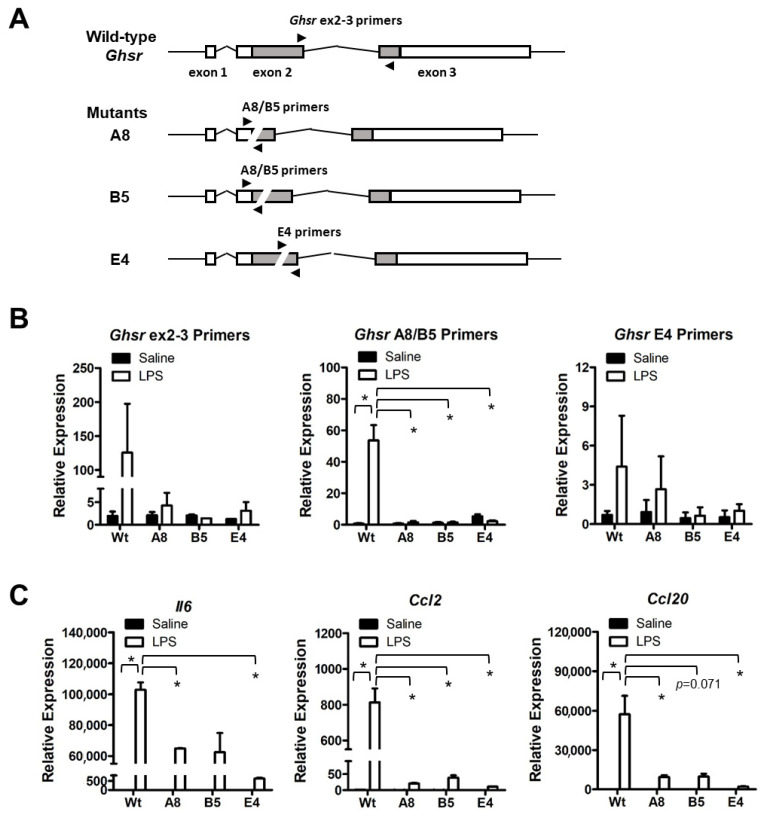
Characterization of RAW264.7 *Ghsr* deletion mutants. (**A**) Wild-type and mutant *Ghsr* genomic structural scheme and qPCR priming sites. (**B**) RAW264.7 cells with wild-type or mutant *Ghsr* were treated with 1 μg/mL LPS for 4 h to induce pro-inflammatory response. LPS stimulation induced *Ghsr* expression in wild-type cells but not in A8, B5 and E4 mutant cells. (**C**) The LPS-induced expressions of pro-inflammatory cytokines and chemokines were drastically reduced in *Ghsr* mutants. Data are reported as mean ± SEM. *: *p* < 0.05.

To further define the function of *Ghsr* in BPA-induced macrophage programming, we generated *Ghsr* deletion mutants in murine macrophage cell line RAW264.7 by Cas9 and guide RNA-mediated gene editing (Figure S1). We isolated three *Ghsr* mutant clones: A8, B5 and E4. Clones A8 and B5 have a partially overlapped deletion region in *Ghsr* exon 2, whereas clone E4 has an independent deletion region in exon 2. All mutants were confirmed by genomic DNA sequencing analysis (Figure S1). In cell culture, we treated RAW264.7 parental cells and mutants with LPS to activate macrophages. Then we examined the differential expression of *Ghsr* by qPCR analysis using the *Ghsr* primers to target the specific regions of deletion in mutants (Figure 6A; Table S2). We found that RAW264.7 parental cells (Wt) increased the expression of *Ghsr* in response to LPS stimulation, but the mutants had no significant response (Figure 6B). Similarly, the mutants had a much lower response to LPS for inducing expressions of pro-inflammatory cytokine *Il6* and chemokines *Ccl2* and *Ccl20* (Figure 6C). The results indicate that *Ghsr* mutants have a functional defect in modulating macrophage activation.

**Figure 7:**
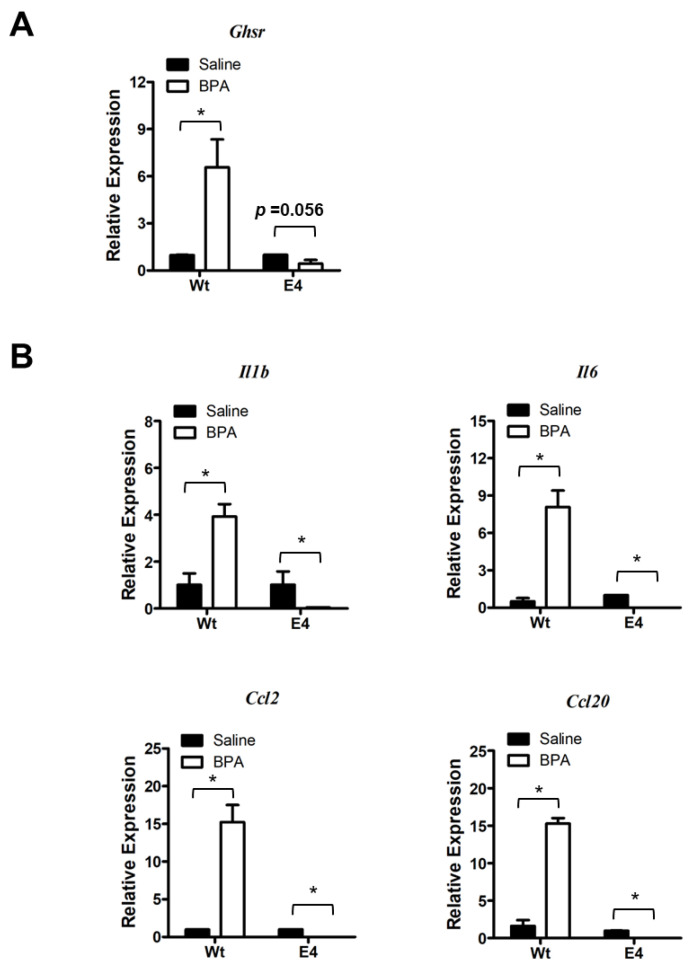
*Ghsr* deletion mutation suppresses the BPA-induced macrophages activation. (**A**) Gene expression analysis showed that BPA induced *Ghsr* gene expression in RAW264.7 parental cells (Wt), but the induction of *Ghsr* expression was suppressed in mutant E4. (**B**) The BPA-induced expressions of pro-inflammatory cytokines *Il1b* and *Il6* and chemokine *Ccl2* and *Ccl20* were blunted by *Ghsr* mutation in mutant E4 cells. Data are reported as mean ± SEM. *: *p* < 0.05.

Following up the mutant characterization, we assessed the BPA response in RAW264.7 parental cells and mutants. We found that *Ghsr* expression was inducible by BPA stimulation in RAW264.7 parental cells (Figure 7A). However, the BPA-induced *Ghsr* expression was greatly suppressed in the mutant E4 cells (Figure 7A). We further examined the BPA-induced pro-inflammatory cytokine and chemokine gene expression and found that the BPA exposure stimulated *Il1b*, *Il6*, *Ccl2*, and *Ccl20* expression in RAW264.7 parental cells (Figure 7B), which was very similar to our observations in colon mucosa (Figure 2) and PM (Figure 5). Remarkably, the BPA-induced expressions of *Ghsr* and pro-inflammatory cytokine/chemokine genes were completely suppressed in mutant E4 (Figure 7A,B). We also performed the gene expression assay in *Ghsr* mutant clone A8 and control cells and found a similar result of the lost response to BPA. To avoid redundancy, we show the results in Figure S2. Our finding suggests that GHSR function is required for BPA-induced pro-inflammatory response in macrophages.

## 4. Discussion

Our study unveiled an important functional relationship between BPA immunotoxicity and ghrelin receptor GHSR function in macrophages. We found that BPA elicits intestinal inflammation in mice. The BPA effect is associated with an enhanced expression of *Ghsr* and the expression of pro-inflammatory cytokines/chemokines in colon mucosa and macrophages. BPA also activates innate immunity systemically, showing markedly increased inflammatory subpopulations of PBMC and PM in blood circulation and the peritoneal cavity. Excessive expression of pro-inflammatory cytokines is characteristic of IBD pathology, which can lead to a disbalanced immune homeostasis and worse prognosis of IBD [41]. Cytokine blockage therapy showed promise in human clinical trials [42] but still largely unmet the need for effective and safe therapeutics of IBD.

We know that BPA is merely one of the representative chemicals in the bisphenol family. Other analogs such as bisphenol S and bisphenol F are used to substitute BPA in manufacture, but they still have similar endocrine disruptor activity [43,44], immunomodulatory effects [45], and the toxicity to cause intestinal inflammation and transcriptomic changes [46,47]. However, the precise molecular mechanism of BPA-like immunotoxicity is still largely ambiguous. In this study, we demonstrated that nutrient-sensing ghrelin receptor GHSR signaling is involved in modulating the BPA effect in macrophages, which provides the possibility of exploring the use of GHSR synthetic antagonists for mitigating the BPA’s and analogs’ detrimental effects in animals and humans.

Previous research has identified certain molecular targets of BPA as being in the estrogen receptors family. Estrogen receptors ERα and ERβ have an affinity to BPA, but the affinity is much lower than estradiol [48,49]. Compared to the binding of ERα, BPA has a relatively high affinity to a membrane-type estrogen receptor, known as G protein-coupled estrogen receptor 1 (GPER1) or G protein-coupled receptor 30 (GPR30) [50]. GPER1 has broad functions in health and disease, including a role in IBD pathophysiology [51]. It has been reported that *GPER1* expresses in human monocytes [52]. To investigate GPER1 status in our study, we examined *Gper1* gene expression in colon mucosa and RAW264.7 macrophage cells. We confirmed that *Gper1* mRNA was expressed in mouse colon mucosa (Figure S3A). In RAW264.7 cells, we also confirmed the *Gper1* expression in both *Ghsr* wild-type and mutant E4 cells (Figure S3B). Therefore, the BPA-induced inflammatory phenotype is likely modulated through GPER1 and GHSR pathway crosstalk.

To recapitulate, the most important finding in our study is that BPA stimulates *Ghsr* expression in macrophages and promotes pro-inflammatory macrophage polarization. It seems that GHSR activity is wired for pro-inflammatory programming in macrophages. We know that GHSR has a high constitutive activity [27], which is associated with Gα_q/11_ protein-coupled inositol phosphate signaling through phospholipase C to mobilize intracellular Ca^2+^ [28]. The elevated calcium signaling leads to the phosphorylation of CREB and in turn the activation of *Il6* and other effector gene transcriptions [53,54]. Therefore, when *Ghsr* expression increases under BPA stimulation, the GHSR—Gα_q/11_ pathway has a higher activity and results in pro-inflammatory phenotypes. This is supported by several lines of evidence in our study. First, the BPA-induced *Ghsr* expression and intestinal inflammation were closely concurrent with an increased expression of *Il6* and *Ccl2* genes in colon mucosa (Figure 2). Second, BPA exposure elevated inflammatory markers TNFα^+^ and CCL2^+^ in PM subpopulations (Figure 4) and also enhanced expression of *Ghsr*, *Il6* and *Ccl2* genes in PM (Figure 5). Third, in the murine macrophage RAW264.7 cell model, when comparing the cells with wild-type *Ghsr* and mutants, the LPS-induced inflammatory effect was drastically suppressed by a loss of *Ghsr* function (Figure 6). Similarly, the BPA-induced expressions of pro-inflammatory cytokines/chemokines *Il1b*, *Il6*, *Ccl2*, and *Ccl20* were clearly abolished in the E4 mutant (Figure 7), suggesting that GHSR is required for a signaling cascade or regulatory pathway that links BPA to downstream effector gene transcription. Our findings warrant further investigation of BPA and GHSR functional mechanisms that may be involved in G protein-coupled receptor heterodimerization or signaling pathway crosstalk in macrophages.

## 5. Conclusions

Our study showed that BPA exposure promotes mouse intestinal inflammation, in part by activating monocytes and macrophages. The BPA effect induces the expression of ghrelin receptor gene *Ghsr* in macrophages. We demonstrated that the expression of *Ghsr* facilitates the BPA-induced pro-inflammatory response. Furthermore, we generated *Ghsr* deletion mutants and conducted functional assays and confirmed that GHSR is required for BPA-induced immunotoxicity in macrophages. Our findings suggest that nutrient-sensing GHSR signaling plays an important role in the modulation of macrophage activity and intestinal inflammation, which may serve as an immune modulator target for combating environmental toxin effects in animals and humans.

## Data Availability

All the relevant data have been provided in the manuscript, and any supplementary datasets used and/or analyzed during the current study are available from the corresponding author on reasonable request.

## Supplementary Materials

The following supporting information can be downloaded at: https://www.mdpi.com/article/10.3390/genes14071455/s1, Table S1: Antibodies used in analysis of mouse PBMC and PM; Table S2: Oligonucleotides used in mouse gene editing, genotyping and qPCR assays. Figure S1: Identification of RAW264.7 *Ghsr* deletion mutants; Figure S2: BPA-induced macrophages activation was suppressed in *Ghsr* mutant clone A8; Figure S3: Expression of *Gper1* in mouse colonic mucosa and murine macrophage RAW264.7.
