# Supplementary material for: Nutrient-Sensing Ghrelin Receptor in Macrophages Modulates Bisphenol A-Induced Intestinal Inflammation in Mice
Source: Genes (Basel). 2023 Jul 16;14(7):1455. doi: 10.3390/genes14071455 (PMC10378756; doi:10.3390/genes14071455)
Supplement: Supplementary file 1 [file genes-14-01455-s001.zip › genes-2497280-supplementary.pdf]

genes

MDPI

*Supplementary materials*

# Nutrient-Sensing Ghrelin Receptor in Macrophages Modulates Bisphenol A-Induced Intestinal Inflammation in Mice

**Xiangcang Ye [1], Zeyu Liu [1], Hyewon Han [1], Jiyeon Noh [1], Zheng Shen [1], Dami Kim [1], Hongying Wang [1], Huiping Guo [2], Johnathan Ballard [2], Andrei Golovko [2], Benjamin Morpurgo [2] and Yuxiang Sun [1,3,*]**

[1] Department of Nutrition, Texas A&M University, College Station, TX 77843, USA
[2] Texas Institute for Genomic Medicine, College Station, TX 77843, USA
[3] USDA/ARS Children's Nutrition Research Center, Department of Pediatrics, Baylor College of Medicine, Houston, TX 77030, USA
* Correspondence: yuxiang.sun@tamu.edu; Tel.: +1-979-862-9143

**Abstract:** Bisphenols are environmental toxins with endocrine disruptor activity, yet bisphenol A (BPA) and its analogs are still widely used in manufacturing plastic products. There is evidence showing that BPA elicits inflammation in humans and animals, but the target cell types of BPA are not well understood. In this study, we sought to determine the BPA's direct effect on macrophages and the BPA immunotoxicity in mouse intestine. Ghrelin is an important nutrient-sensing hormone, acting through its receptor growth hormone secretagogue receptor (GHSR) to regulate metabolism and inflammation. We found that BPA promotes intestinal inflammation, showing increased infiltrating immune cells in colons and enhanced expression of *Ghsr* and pro-inflammatory cytokine and chemokine, such as *Il6* and *Ccl2*, in colonic mucosa. Moreover, we found that both long- and short-term BPA exposure elevated pro-inflammatory monocytes and macrophages in mouse peripheral blood mononuclear cells (PBMC) and peritoneal macrophages (PM), respectively. To determine the role of GHSR in BPA-mediated inflammation, we generated *Ghsr* deletion mutation in murine macrophage RAW264.7 using CRISPR gene editing. In wild-type RAW264.7 cells, the BPA exposure promotes macrophage pro-inflammatory polarization and increased *Ghsr* and cytokine/chemokine *Il6* and *Ccl2* expression. Interestingly, *Ghsr* deletion mutants showed a marked reduction of pro-inflammatory cytokine/chemokine expression in response to BPA, suggesting that GHSR is required for the BPA-induced pro-inflammatory response. Further understanding how nutrient-sensing GHSR signaling regulates BPA intestinal immunotoxicity will help design new strategies to mitigate BPA immunotoxicity and provide policy guidance for BPA biosafety.

**Keywords:** ghrelin; GHSR; bisphenol A; macrophage; inflammation

**Table S1.** Antibodies used in analysis of mouse PBMC and PM

| Panel | Ab Name | Fluorophore | Catalog |
|---|---|---|---|
| PBMC panel | | | |
| | anti-CD45 | eFluor450 | Thermo 48-0451-82 |
| | anti-F4/80 | PE-Cy7 | Thermo 25-4801-82 |
| | anti-CD11b | APC-Cy7 | BD 561039 |
| | anti-CD38 | BV750 | BD 747103 |
| | anti-Ly6G | PerCP | Biolegend 127654 |
| | anti-CD206 | BV650 | Biolegend 141723 |
| | anti-iNOS | AF488 | Thermo 53-5920-82 |
| PM panel | | | |
| | anti-CD45 | BV510 | Biolegend 103138 |
| | anti CD11b | PE | Biolegend 101208 |
| | anti Ly6C | eFluor-450 | Thermo 48-5932-82 |
| | anti-Ly6G | BV785 | Biolegend 127645 |
| | anti-CD115 | APC | Biolegend 135509 |
| | anti-CX3CR1 | BV650 | Biolegend 149033 |
| | anti-CCR2 | FITC | Biolegend 150607 |

**Table S2.** Oligonucleotides used in mouse gene editing, genotyping and qPCR

| Oligo Type | Name | Sequence (5′ --> 3′) | Note |
|---|---|---|---|
| Guide RNAs | | | |
| | gRNA1 | GUGGAACGCGACGCCCAGCG | Target *Ghsr* at coding aa 7-8 |
| | gRNA2 | CGGCACUCGUUGGUGUCCCG | Target *Ghsr* at coding aa 192-193 |
| Genotyping primers | | | |
| | Ghsr-F | CTCCTCAGGGGACCAGATTT | 740 bp (wt) or 150-700 bp (CRISPR del) |
| | Ghsr-R | GAGCACAGTGAGGCAGAAGA | |
| Gene expression primers | | | |
| | Ghsr-F1123 | AAGATGCTTGCTGTGGTGGT | Priming *Ghsr* exons 2 - 3 (wt) |
| | Ghsr-R1284 | AGCGCTGAGGTAGAAGAGGA | |
| | Ghsr A8/B5-F112 | CTCAGGGGACCAGATTTCCG | Priming a Ghsr A8/B5 deletion region |
| | Ghsr A8/B5-R261 | GCAGCAGTTCGTCAGAGAGT | |
| | Ghsr E4-F684 | CCCATCTTCGTGCTGGTG | Priming a Ghsr E4 deletion region |
| | Ghsr E4-R941 | ACCACCACAGCAAGCATCT | |
| | Ghrl-F248 | AGCTGGAGATCAGGTTCAATGC | Priming *Ghrl* exons 2 - 3 |
| | Ghrl-R453 | GCTGAGGCGGATGTGAGTTC | |
| | Gper1-F372 | ACACTCACACACTCTGGGTGC | |
| | Gper1-R607 | TTCCCTCGGCAGTTTTCAGG | |
| | Il1b-F149 | TGTTCTTTGAAGTTGACGGACCC | |
| | Il1b-F476 | TCATCTCGGAGCCTGTAGTGC | |
| | Il6-F162 | ACAAGTCCGGAGAGGAGACT | |
| | Il6-R299 | GAATTGCCATTGCACAACTCT | |
| | Ccl2-F177 | CACTCACCTGCTGCTACTCA | |
| | Ccl2-R293 | GCTTGGTGACAAAAACTACAGC | |
| | Ccl20-F87 | TCCTTGCTTTGGCATGGGTA | |
| | Ccl20-R156 | CAGTCGTAGTTGCTTGCTGCTTC | |
| | Actb-F30 | ACTGTCGAGTCGCGTCCA | |
| | Actb-R117 | TCATCCATGGCGAACTGGTG | |
| | Ppia-F351 | GCTGGACCAAACACAAACGG | |
| | Ppia-R422 | ATGCTTGCCATCCAGCCATT | |

A

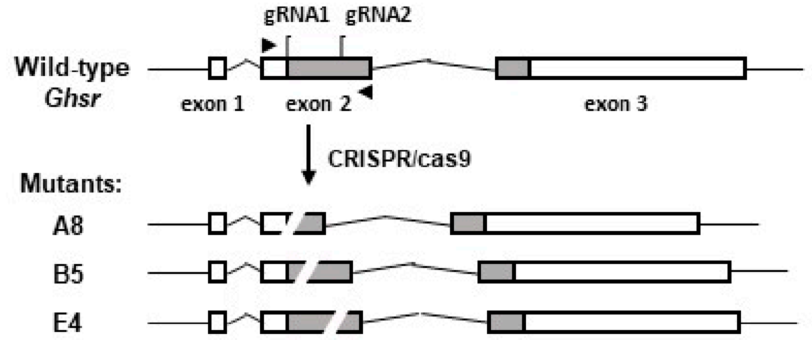


B

| Genotyping Primers: | |
|---|---|
| F/R = 740 bp (wt) + 150-700 bp (del) | |
| Ghsr F | CTCCTCAGGGGACCAGATTT |
| Ghsr R | GAGCACAGTGAGGCAGAAGA |

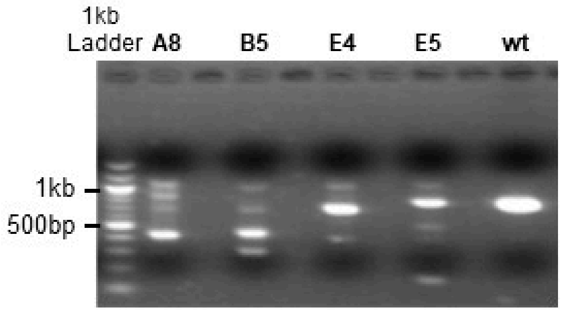


C

| Clone name | PCR results | Sequencing results |
|---|---|---|
| A8 | dominant mutation band around 400bp | deletion aa1-119:<br>GTGGAACGCGACGCCCAGCGAGGAGCCGGAGCCTAACGTCACGCTGGACCTGGA<br>CTGGGACGCTTCTCCCGGCAACGACTCACTCTCTGACGAACTGCTGCCACTGTT<br>CCCCGCGCCGCTGCTGGCGGGCGTCACTGCCACCTGCGTGGCGCTCTTCGTGGT<br>GGGCATCTCGGGCAACCTGCTCACCATGCTGGTGGTGTCCCGCTTCCGCGAGCT<br>GCGCACCACCACCAACCTCTACCTATCCAGCATGGCCTTCTCCGATCTGCTCAT<br>CTTCCTGTGCATGCCGCTGGACCTCGTCCGCCTCTGGCAGTATCGGCCCTGGAA<br>CTTCGGCGACCTGCTCTGCAAACTCTTCCAG |
| B5 | dominant mutation band around 400bp, also weaker 300 bp | deletion aa4-110:<br>CGACGCCCAGCGAGGAGCCGGAGCCTAACGTCACGCTGGACCTGGACTGGGACG<br>CTTCTCCCGGCAACGACTCACTCTCTGACGAACTGCTGCCACTGTTCCCCGCGC<br>CGCTGCTGGCGGGCGTCACTGCCACCTGCGTGGCGCTCTTCGTGGTGGGCATCT<br>CGGGCAACCTGCTCACCATGCTGGTGGTGTCCCGCTTCCGCGAGCTGCGCACCA<br>CCACCAACCTCTACCTATCCAGCATGGCCTTCTCCGATCTGCTCATCTTCCTGT<br>GCATGCCGCTGGACCTCGTCCGCCTCTGGCAGTATCGGCCCTGGAACTTC |
| E4 | dominant mutation band around 700bp | deletion aa181-197<br>GTGGGCGTGGAGCACGAGAACGGCACAGATCCCCGGGACACCAACGAGTGCCGC<br>GCCACCGAGTTCGCTG |
| E5 | wt band 740bp and weak mutant 150 bp band | no mutations found |
| wt RAW264.7 | wt band 740bp | |

**Figure S1. Generation of RAW264.7 *Ghsr* deletion mutants by CRISPR gene editing.** (**A**) Two gRNAs targeted to Ghsr exon 2 for cas9-mediated deletion. (**B**) The isolated RAW264.7 cell clones were firstly checked by genotyping PCR, which primers were flanked the gRNAs targeting region in exon 2. (**C**) The genotyping identified clones were further confirmed by DNA sequencing.

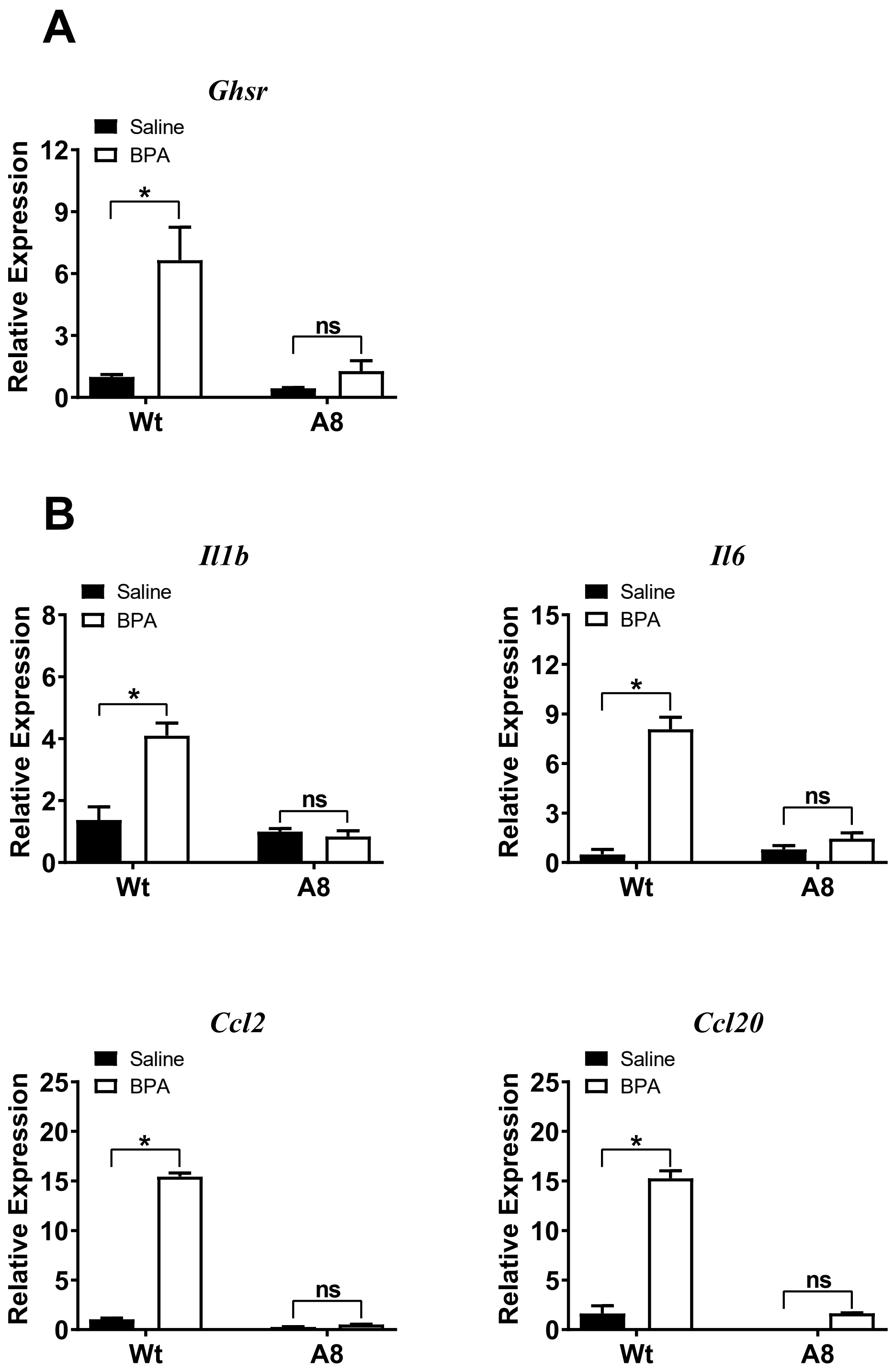


**Figure S2. BPA-induced macrophages activation was suppressed in *Ghsr* mutant clone A8.** (**A**) Gene expression analysis showed that BPA induced *Ghsr* gene expression in RAW264.7 parental cells (Wt), while the induction of *Ghsr* expression was suppressed in mutant A8. (**B**) The BPA-induced expressions of pro-inflammatory cytokines *Il1b* and *Il6* and chemokine *Ccl2* and *Ccl20* were blunted by *Ghsr* mutation in mutant A8 cells. Data are reported as mean ± SEM. *: $p < 0.05$; ns: not significant

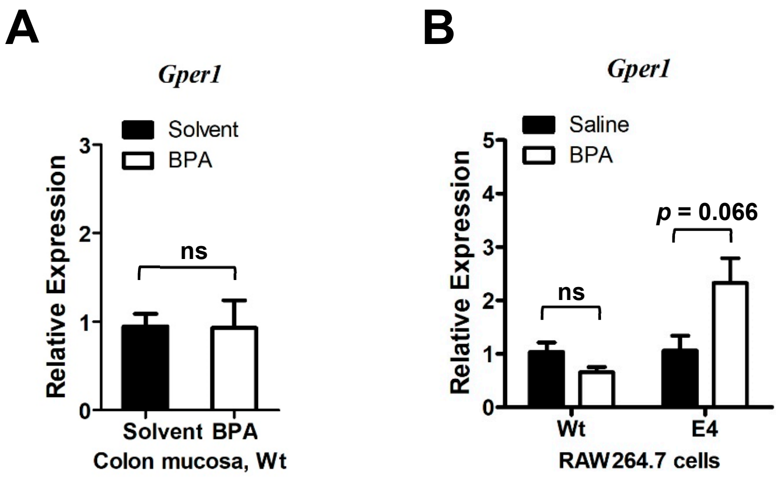


**Figure S3. Expression of *Gper1* in mouse colonic mucosa and RAW264.7 cells.** (**A**) The expression of *Gper1* in mouse colonic mucosa was analyzed by qPCR assay, showing little changes in *Gper1* expression influenced by BPA stimulation. (**B**) RAW264.7 cells also express *Gper1*, which is not significantly affected by *Ghsr* mutation. Mouse group n = 4 in colon mucosa samples. Data are reported as mean ± SEM. ns: not significant.
